# The Hepatitis C Virus Glycan Shield and Evasion of the Humoral Immune Response

**DOI:** 10.3390/v3101909

**Published:** 2011-10-14

**Authors:** François Helle, Gilles Duverlie, Jean Dubuisson

**Affiliations:** 1 Laboratory of Virology, EA4294, Jules Verne University of Picardie, Amiens 80000, France; E-Mail: gilles.duverlie@u-picardie.fr; 2 Virology Department, Amiens University Hospital Center, South Hospital, Amiens 80000, France; 3 Inserm U1019, CNRS UMR8204, Center for Infection and Immunity of Lille (CIIL), Institut Pasteur de Lille, Université Lille Nord de France, Lille 59021, France; E-Mail: jean.dubuisson@ibl.fr

**Keywords:** hepatitis C virus, neutralizing antibodies, viral escape, *N*-glycosylation

## Abstract

Despite the induction of effective immune responses, 80% of hepatitis C virus (HCV)-infected individuals progress from acute to chronic hepatitis. In contrast to the cellular immune response, the role of the humoral immune response in HCV clearance is still subject to debate. Indeed, HCV escapes neutralizing antibodies in chronically infected patients and reinfection has been described in human and chimpanzee. Studies of antibody-mediated HCV neutralization have long been hampered by the lack of cell-culture-derived virus and the absence of a small animal model. However, the development of surrogate models and recent progress in HCV propagation *in vitro* now enable robust neutralization assays to be performed. These advances are beginning to shed some light on the mechanisms of HCV neutralization. This review summarizes the current state of knowledge of the viral targets of anti-HCV-neutralizing antibodies and the mechanisms that enable HCV to evade the humoral immune response. The recent description of the HCV glycan shield that reduces the immunogenicity of envelope proteins and masks conserved neutralizing epitopes at their surface constitutes the major focus of this review.

## 1. Introduction

The hepatitis C virus (HCV) is a major public health problem worldwide. More than 170 million people worldwide are seropositive for HCV and thus risk developing cirrhosis and hepatocellular carcinoma [1]. Indeed, HCV tropism is principally restricted to the liver and the HCV viral cycle has been shown to be tightly linked to the hepatocyte’s lipid metabolism. In particular, HCV particle production depends on assembly and secretion of very low-density lipoproteins and plasma-derived HCV particles have been reported to be in complex with low and very low-density lipoproteins [2–7]. However, the nature of the association between HCV and these lipoproteins remains unclear [8].

Today’s standard treatment for HCV infection is combination therapy with pegylated interferon and ribavirin [9]. However, this therapy is expensive, relatively toxic and effective in only half of treated patients. Specific, directly acting anti-HCV drugs are now entering the market and will hopefully soon provide substantial improvements over current treatments [10]. The development of a protective vaccine against HCV has proven to be extremely challenging but is still being pursued, since it would constitute the most cost-effective means to reduce HCV spread to uninfected individuals. Extensive research in this area suggests that a successful HCV vaccine will need to stimulate: (i) the production of antibodies (Abs) that exhibit antiviral activity (also referred to as neutralizing Abs (NAbs)); and (ii) potent HCV-specific T cell responses. To this end, it is essential to define all the neutralizing determinants displayed by HCV envelope glycoproteins and particularly conserved structures that could enable cross-neutralization between diverse virus genotypes and minimize the likelihood of immune escape. It is also important to understand the molecular basis of HCV resistance to neutralization.

Despite the induction of effective immune responses, 80% of HCV-infected individuals progress from acute to chronic hepatitis. Spontaneous viral clearance occurs in approximately 20% of acutely infected individuals and results in the resolution of the infection without sequelae. It is believed that the type and strength of the host immune responses during the acute phase of HCV infection determine the outcome. The importance of CD4 and CD8 T cells in clearing HCV infection is widely accepted. In contrast, the role played by Abs in HCV clearance remains subject to debate. Infection by HCV induces the production of Abs against various HCV proteins in the majority of chronically infected people. Moreover, NAbs have been detected in the sera of HCV-infected patients [11–17]. These NAbs may be classified as isolate-specific or cross-neutralizing, depending on their ability to neutralize only the autologous virus or heterologous viral strains.

Studying the relative contribution of Abs to HCV clearance has long been hampered by the lack of convenient *in vitro* models for evaluating the neutralizing activity of anti-HCV Abs. However, the development of retroviral particles pseudotyped with HCV envelope proteins (HCVpp) [12, 18, 19] and cell culture-derived HCV (HCVcc) [20–22] now enable sensitive and robust neutralization assays to be performed. Although HCVpp do not mimic all the complex features of native viral particles [23–28], *in vitro* neutralization in the HCVpp model system usually correlates well with neutralization of infectious HCVcc. Importantly, the very recent development of an immunocompetent, genetically humanized mouse model, which recapitulates a part of the HCV life cycle, is opening up new opportunities for studying HCV neutralization *in vivo* [29].

Recent studies suggest that rapid induction of NAbs during the early phase of infection may help clear or control HCV infection [14,30,31]. However, doubt has been cast on the role of NAbs in host protection, since: (i) HCV is able to escape NAbs in chronically infected patients; and (ii) reinfection has been described in both humans and chimpanzees [32–34]. Mechanisms that enable HCV to evade the humoral immune response are starting to be elucidated and form the theme of this review. Here, we summarize recently accumulated knowledge on the viral targets of anti-HCV NAbs and anti-HCV NAbs escape strategies, with a special focus on our recent findings concerning the HCV glycan shield.

## 2. HCV Envelope Glycoproteins

### 2.1. HCV Envelope Glycoproteins and Viral Entry

**Figure 1.**
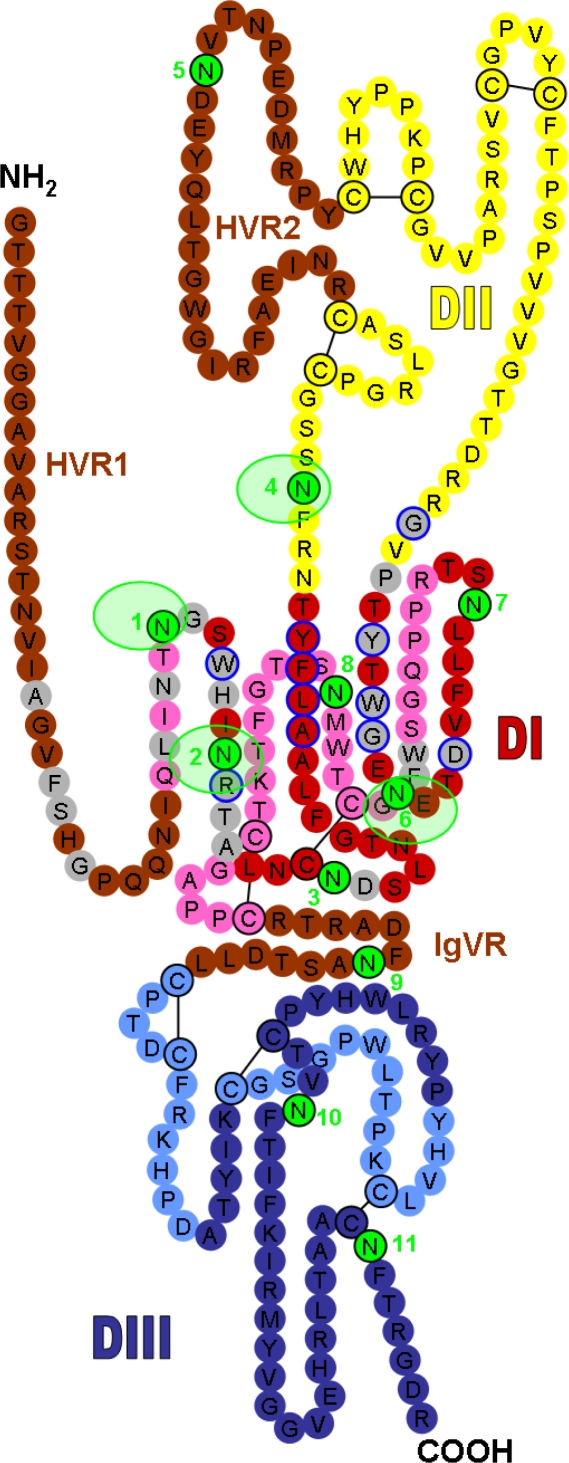
Localization of the N-linked glycans on the model of hepatitis C virus (HCV) E2 glycoprotein (modified version of the figure published by Helle *et al.* [28], adapted from the model recently published by Krey *et al.* [37]). The linear sequence of the JFH-1 strain E2 ectodomain without the stem region is represented as a chain of beads (colored circles) labeled with the corresponding amino acid and threaded onto a class II fold. The three putative domains are presented in red (DI), yellow (DII), and blue (DIII), and the variable regions (HVR1, HVR2, and IgVR) are indicated in brown. Circles in pale and bright colors represent residues in the background and foreground of the domains, respectively. Disulfide bonds are indicated by black bars. DI domain residues that are involved in CD81 binding [48] are outlined in blue. Amino acids recognized by known anti-HCV monoclonal neutralizing antibodies (NAbs) (Table 1) are shown as grey circles. Glycosylation sites are shown as sequentially-numbered green circles. Glycosylation sites masking the CD81 binding site are highlighted with light green shading.

HCV is a small, enveloped, single-stranded positive RNA virus that belongs to the *Hepacivirus* genus within the *Flaviviridae* family and infects only humans and chimpanzees *in vivo* [1]. This virus displays a high degree of genetic heterogeneity and has been classified into seven genotypes and several subtypes. Its genome encodes a single polyprotein precursor of about 3,000 amino acid residues, which is cleaved co- and post-translationally by host and viral proteases to yield ten mature products [1]. The two envelope glycoproteins, E1 and E2, are released from the polyprotein by signal peptidase cleavages. These glycoproteins are type I membrane proteins with a C-terminal transmembrane domain anchored in the lipid envelope. These two proteins assemble as non-covalent heterodimers, which are mainly retained in the endoplasmic reticulum [35], and they are found as large disulfide-linked oligomers on the surfaces of HCV particles [36]. A high-resolution structure of HCV envelope proteins is still lacking but a schematic representation of the three-dimensional organization of E2, predicted by disulfide mapping and molecular modeling, was published recently [37]. This model proposes that the ectodomain is composed of three domains (Domains I, II and III) followed by a stem region (Figure 1). Interestingly, functional studies have recently confirmed the bipartite composition of Domain I suggested by this model [27].

The HCV envelope glycoproteins E1 and E2 play an important role in the binding step of the entry process [38]. Indeed, HCV attaches to host cells via interactions between E1E2 and several cellular entry factors. Some studies suggest that glycosaminoglycans may serve as the initial docking site for HCV [39,40]. Although it has been suggested that the envelope proteins play a role in this interaction, involvement of the HCV-associated lipoproteins in the initial glycosaminoglycan binding cannot be ruled out [41]. In view of the association between HCV and lipoproteins, the LDL receptor has also been suggested as another potential attachment factor for HCV [42–44]. However, the role of this receptor in HCV entry remains unclear. After the initial attachment to the host cell, a virus generally binds to specific entry factors that are responsible for initiating a series of events leading to release of the viral genome into the cytosol. Several cell surface proteins have been described as specific entry factors for HCV and interactions with these molecules do appear to occur in a programmed series of events. The first identified and best characterized entry factor is the tetraspanin CD81, which was initially shown to interact with HCV glycoprotein E2 [45] (for a review, see [46]). Several E2 residues involved in the CD81 interaction have been identified (Figure 1) [47–49].

Following the identification and characterization of CD81 as a molecule involved in HCV entry, HCV glycoprotein E2 was found to also interact with the human scavenger receptor class B type I (SR-BI, also referred to as CLA-1). Hence, SR-BI has also been suggested as a potential entry factor for HCV [64,65]. It appears that HCV exploits SR-BI physiological functions during the entry process (for a review, see [66]). The kinetics of infection inhibition with anti-SR-BI- and anti-CD81-Abs suggest that SR-BI is involved in virus/cell recognition upstream of the CD81 interaction [67,68]. Indeed, it is likely that the HCV particle encounters SR-BI before CD81, since it can bind to CHO cells expressing SR-BI but not to CHO cells expressing CD81 [69]. The hypervariable region 1 (HVR1) in E2 is important for interaction with SR-BI [64,70,71] and it has recently been suggested that HVR1 masks the CD81 binding region [71]. Thus, initial contact with SR-BI may be needed to unmask the CD81 binding region on E2 and thus enable the particle to interact with CD81. Although a direct interaction between HVR1 and SR-BI could take place, it has also been suggested that the lipoproteins associated with the viral particle interact with this entry factor [72]. Indeed, SR-BI is also a receptor for low-density lipoproteins [73]. Whether this indirect interaction plays a role in HCV entry, however, remains to be determined.

Recently, the tight junction proteins Claudin-1 (CLDN1) and Occludin (OCLN) have been identified as additional entry factors for HCV [69,74]. CLDN6 and 9 are also able to mediate HCV entry [75,76]. The interaction between CLDN and CD81 seems to be important for HCV entry process [77–79] and is regulated by receptor tyrosine kinases EGFR and EphA2 [80]. Furthermore, indirect E2-OCLN association was demonstrated in co-immunoprecipitation and pull-down assays [81,82]. However, a direct interaction between CLDN or OCLN molecules and HCV envelope glycoproteins has not yet been reported [83]. Thus, the precise role of CLDN and OCLN proteins in HCV entry remains to be determined.

HCV enters target cells via clathrin-mediated endocytosis [84] and it has been suggested that fusion occurs in the early endosomes [85]. The endosomes acidic pH has been shown to trigger the fusion process, probably by inducing conformational changes in the envelope proteins [19,40,70,84–86]. The precise roles of E1 and E2 in the fusion step have not yet been determined. It has been suggested that amino acids 262–290 in E1 as well as 416–430, 502–520 and 600–620 in E2 play a role in the fusion process and it could be that both proteins are involved in this process [37,87–94]. It has also been reported that E2 can bind lipid membranes devoid of proteins after acidic treatment, which suggests a direct role for this protein in the fusion process [95]. Interestingly, the transmembrane domains of HCV envelope glycoproteins also play an active role in the fusion process. Indeed, mutations in these domains affect the fusion properties of HCV envelope glycoproteins, possibly by affecting the oligomeric reorganization of the fusion protein [96]. After fusion between the viral envelope and an endosomal membrane, the viral genome is released into the cytosol.

Interestingly, it was recently reported that the host neutralizing responses in HCV-infected patients target viral entry after HCV binding and are most likely related to HCV-CD81 and HCV-SR-BI interactions or membrane fusion [51].

### 2.2. Neutralizing Determinants in HCV Envelope Glycoproteins

The NAb major target is the E2 envelope glycoprotein. The first neutralizing epitopes on HCV envelope glycoproteins to be described were located within HVR1 [97]. Statistical analyses have suggested the presence of two immunogenic domains in HVR1, encompassing the N-terminal part and the C-terminal part, respectively [98]. However, anti-HVR1 Abs are often partially conformation-sensitive [55–57]. Data obtained with various anti-HVR1 Abs suggest that the C-terminal region is the main neutralization determinant in HVR1 [13,19,54,56,57]. Indeed, the rat anti-HVR1 monoclonal Abs (mAbs) 6/16, 7/59 and 6/82, which bind to the N-terminal part of HVR1, do not neutralize HCV infectivity [19]. In contrast, mAbs 9/27, 3C7, J6.36 and AP213, as well as the polyclonal Abs R140 and R1020, whose target epitopes include the C-terminal part of HVR1, neutralize HCV infection [13,19,54,56,57]. Detailed mapping identified residues at positions 400, 403, 404 and 406 as key epitope residues for J6.36, AP213, R140 and R1020 (Figure 1 and Table 1) [56,57]. Abs directed against HVR1 may neutralize HCV by blocking the interaction between E2 and SR-BI, as described for the 9/27 and J6.36 mAbs [57,64]. However, while there is strong evidence to suggest that NAbs directed against HVR1 correlate with a beneficial outcome, these Abs usually present limited cross-reactivity.

In contrast, a number of groups have pointed out the potential existence of additional neutralizing epitopes elsewhere in the E2 glycoprotein by describing Abs with a broader neutralizing activity [13,16,99]. In particular, several discontinuous regions of E2 contain highly conserved residues involved in CD81 binding and are targeted by NAbs [48]. Several studies have described monoclonal NAbs directed against these broadly conserved epitopes in different HCV genotypes [57,58,100]. For instance, the mouse AP33 mAb and the rat 3/11 mAb have broad neutralizing activity that can be attributed to the extreme conservation of their epitopes and the importance of the targeted region in CD81 binding [57,58,100]. Human mAbs recognizing conserved neutralizing epitopes on E2 envelope proteins, that are of greater interest for the development of therapeutic strategies, have also been described by several groups [23,60,63,101–106]. Detailed mapping and selection of escape variants identified residues at positions 415, 420, 424, 523, 525, 529, 530 and 535 as key residues involved in the corresponding neutralizing epitopes (Figure 1 and Table 1) [23,57–63,105,107]. Many of these residues are also important for E2 binding to CD81 (Figure 1) [47–49]. This cluster of overlapping neutralizing epitopes, referred to as “domain B” by Steven Foung’s group and located in Domain I of the HCV E2 structural model (Figure 1), is conserved across most HCV genotypes and thus is an attractive target for vaccine design.

E1-specific NAbs have also been described but are rare, probably due to the poor immunogenicity of this protein or the immunodominance of E2. An E1-specific serum has been shown to neutralize HCVpp and HCVcc [53,108]. Furthermore, three human mAbs recognizing neutralizing epitopes on E1 have been described: H-111, which has moderate neutralizing activity [52,53], IGH520 (a sister clone of IGH505) and IGH526, which neutralize numerous HCV genotypes [50,51]. This shows that neutralizing epitopes in E1 are targeted by host responses *in vivo*. In particular, the H-111 epitope is located in the amino-terminal portion of E1 (amino acids 192–202; Table 1) [52]. Furthermore, Haberstroh *et al.* showed that the region corresponding to amino acids 313–326 is targeted by IGH520 and IGH526 during postbinding events (Table 1) [51].

## 3. Evasion of the Humoral Immune Response

The ability of HCV to persist in its host in the presence of NAbs has yet to be explained. Several mechanisms by which HCV could evade the host humoral immune response have been suggested (see [109] for a detailed review). It is thought that the high variability of HCV genomic RNA represents a first escape strategy. Typically, the presence of different but closely related viral variants within the same individual (commonly defined as “quasispecies”) may allow the virus to circumvent the immune response [110–113]. However, it has long been difficult to assay NAbs activity against viral variants present in patient sera at the time of sample collection. Two recent studies looked at envelope glycoprotein sequence evolution and neutralization of sequential autologous HCVpp and found that HCV continuously escapes the host neutralizing response by mutations resulting in loss of NAb binding to HCV envelope glycoproteins [31,114]. This finding suggests that the host NAb response lags behind the rapidly evolving HCV envelope glycoprotein sequences in the quasispecies population. To be more precise, several studies have demonstrated that envelope gene evolution, particularly in the HVR1, is shaped by NAb pressure and occurs as a direct response to immune pressure from NAbs [31,114–116]. Interestingly, it has been observed that HVR1 can remain stable for up to 21 months without NAb pressure but shifts from its initial sequence after initiation of a NAb response [116]. Importantly, the infection outcome in humans can be predicted by sequence changes in E2 HVR1 [117]. Recently, it has also been suggested that HVR1 obstructs the viral CD81 binding site on E2 and decreases the exposure of crucial conserved epitopes, thus preventing effective neutralization [71,118]. Thus, HVR1 may act as an immunological decoy that diverts the immune system and shields conserved neutralizing epitopes. The lipoproteins associated with the virions could also protect HCV against NAbs [2,72,119,120]. In particular, a correlation was observed between HCVcc density and sensitivity to neutralizing immunoglobulin G, suggesting that lipoproteins reduce the sensitivity of particles to NAbs [119]. Furthermore, high-density lipoproteins have also been shown to attenuate the neutralization of HCVpp by Abs from HCV-infected patients by accelerating HCV entry [53,121,122]. Cell-to-cell transmission could also prevent HCV from being recognized by NAbs once an infection is established [123–126]. Lastly, the presence of interfering Abs has also been described [127,128]. These Abs disrupt virus neutralization mediated by Abs recognizing residues 412–426 on E2 by binding to non-neutralizing epitopes at residues 434–446.

## 4. The HCV Glycan Shield

The ectodomains of HCV envelope glycoproteins are highly glycosylated. E1 contains four conserved N-glycosylation sites (E1N1 to E1N4; positions 196, 209, 234 and 305 in the H77 strain) [129]. Other sites are conserved in only some genotypes: position 250 in genotypes 1b and 6 and position 299 in genotype 2b. Nine glycosylation sites in E2 are conserved across all genotypes (E2N1, E2N2, E2N3, E2N4, E2N6, E2N8, E2N9, E2N10 and E2N11; positions 417, 423, 430, 448, 532, 556, 576, 623 and 645 in the H77 strain) (Figure 1) [129]. A site at position 476 (E2N5) exists in most genotypes but is rarely present in genotype 1b sequences. Another site at position 540 (E2N7) is absent in genotypes 3 and 6. Thus, despite variability in HCV envelope glycoprotein sequences, most E1E2 N-glycosylation sites are highly conserved, suggesting that the glycans associated with these proteins play an essential role in the HCV life cycle. Importantly, all these sites have been shown to be modified by glycans [130]. Potential O-glycosylation sites have also been predicted on the E2 envelope protein [131]. However, mass spectrometry analysis of the E2 ectodomain did not reveal the presence of O-glycans [132].

Site-directed mutagenesis in HCVpp or HCVcc systems has enabled researchers to study the functional role of the N-glycans associated with HCV envelope proteins (see Table 2 for a summary). The results indicate that several glycans have an important role in virion assembly and infectivity [28,130,131]. Indeed, mutation of glycosylation sites E1N1, E2N8 or E2N10 leads to envelope protein instability and virion assembly defects [28]. Interestingly, these mutations also lead to a decrease in recombinant E1E2 heterodimerization and affect the incorporation of envelope proteins at the HCVpp surface [130]. These results indicate that glycans E1N1, E2N8 and E2N10 are important for E1E2 folding and heterodimerization and thus for virion assembly. Surprisingly, mutation of glycosylation site E1N4, which also leads to a decrease of recombinant E1E2 stability, heterodimerization and incorporation on HCVpp [130], had only a slight effect on HCVcc infectivity [28].

Glycans associated with viral envelope proteins can modulate the latter’s entry functions by modifying the affinity for one or more receptors or by affecting fusion activity. It has been observed that the loss of glycan at position E2N6 increases the infectivity of HCVcc [28]. Furthermore, this mutant is also more sensitive to inhibition by a soluble form of the CD81 large extracellular loop. Lastly, it has been shown that a soluble form of E2 lacking glycan at position E2N6 exhibits a higher affinity for CD81 than the native molecule [131]. Overall, these data suggest that the better fitness of E2N6 mutant *in vitro* is due to a stronger interaction with CD81. Interestingly, the emergence of adaptive mutations leading to the loss of the E2N6 glycosylation site has been observed in cell culture [133,134]. In contrast, mutation of the E2N7 glycosylation site leads to a strong decrease in HCVcc infectivity but does not affect viral particle secretion [28]. Thus, it seems that glycan E2N7 is located in a key region of E2 that modulates viral entry. However, this modulation is probably genotype-specific since the site is absent in genotypes 3 and 6. The observed differences between genotypes 1a and 2a HCVpp agree with this hypothesis [28,130,131].

It is noteworthy that for some mutants, the envelope glycoprotein entry functions are differently affected by glycan loss in HCVcc and HCVpp systems [28]. In particular, mutation of the E2N2 or E2N4 glycosylation sites had only a slight effect on HCVcc infectivity, whereas the same mutations led to the secretion of non-infectious HCVpp. These discrepancies are probably due to assembly process differences between HCVpp and HCVcc [3,135], which could be responsible for differences in glycan processing [36] and/or differences in the organization of HCV envelope proteins at the surface of the particle [35,36]. Lastly, an effect of HCVcc-associated lipoproteins on the properties of the envelope proteins cannot be ruled out [3,136]. Differences between the entry functions of HCV envelope glycoproteins in HCVpp and HCVcc have been reported elsewhere [23–27].

Interestingly, we recently used the HCVcc system to demonstrate that at least five glycans on E2 (E2N1, E2N2, E2N4, E2N6 and E2N11) reduce HCVcc sensitivity to neutralization. This indicated that glycans limit the recognition of neutralizing epitopes at the surface of E2 (Figure 1) [28]. Indeed, the absence of one of these glycans leads to a higher sensitivity to neutralization by Abs purified from the sera of HCV seropositive patients, as well as mAbs. These data are in agreement with those obtained in the HCVpp system for E2N1, E2N6 and E2N11 mutants [129,131]. The E2N2 and E2N4 mutant sensitivity to neutralization could not be tested in the HCVpp system, since the corresponding mutations lead to the production of non-infectious pseudoparticles. Interestingly, our results suggest that neutralizing epitopes located in HVR1 are not masked by E1E2 associated glycans, since the mutation of glycosylation sites did not modify the sensitivity of HCVpp to neutralization with mAbs 9/27 and 3C7 directed against this region [129]. In contrast, glycans E2N1, E2N2, E2N4, E2N6 and E2N11 modulate the neutralizing activity of mAbs directed against conserved epitopes. In addition, E2N1, E2N2, E2N4 and E2N6 also modulate the inhibition of HCV infectivity by a soluble form of CD81, suggesting that the CD81 binding site on E2 is the NAb target that is protected by glycans. It remains to be determined whether the glycans and HVR1 shielding effects for the CD81 binding site are additive. To answer this question, it would be useful to test the sensitivity of the corresponding mutants in the context of HVR1-deleted HCV.

Using HCVpp and purified Abs from HCV-seropositive patients, we did not observe any effect of E1 glycosylation mutations on sensitivity to neutralization [129]. This suggests that either neutralizing epitopes on E1 are not protected by glycans or the neutralizing immune response against HCV is dominated by anti-E2 Abs. To discriminate between these two possibilities, it would be interesting to study the sensitivity of E1 glycosylation mutants to anti-E1 neutralizing mAbs. In particular, glycans E1N1 and E1N2, located in the region recognized by mAb H-111, are not required for Ab binding, since successful competition for binding with synthetic peptides has been demonstrated. However, whether a lack of these glycans increases sensitivity to neutralization has not been yet determined.

In addition to modulating the accessibility of the CD81 binding region, HCV glycans could also reduce access to other protein regions. Indeed, one third of the molecular mass of E1E2 heterodimers corresponds to glycans. Thus, it is likely that the presence of glycans at the surface of HCV particles limits the immunogenicity of the envelope proteins. The observation that the anti-E1 humoral immune response is stronger after mutation of glycosylation site E1N4 argues in favor of this hypothesis [137, 138]. It has not yet been determined whether the glycans that protect the CD81 binding region from NAb recognition also limit the immunogenicity of this region. It would be interesting to see whether the use of the E2 glycosylation mutants, rather than fully glycosylated E2, could enhance the production of NAbs. Thus, in addition to the mechanisms described in the previous section, the presence of glycans at the surface of HCV envelope proteins could help explain how HCV evades the humoral immune response and why most HCV infections lead to chronicity.

## 5. Conclusions and Perspectives

In conclusion, recent research has provided much insight into HCV-neutralizing epitopes and the virus ability to evade the humoral immune response. Several data evidence that HCV envelope protein N-glycans mask conserved neutralizing epitopes at the surface of HCV particles and also limit envelope protein immunogenicity. Many viruses that impact human health, including HIV and influenza, use glycosylation for immune evasion, since glycans are synthesized by the host cells and are basically self-structures [139]. HIV is one of the most studied viruses with respect to glycosylation and its gp120 envelope protein is one of the most heavily glycosylated proteins in nature, with an average of 25 potential N-linked glycosylation sites (range: 18–33) [140]. Hence, glycosylation accounts for roughly 50% of gp120 molecular mass. Free gp120 glycoproteins have three antigenic faces: (i) the non-neutralizing face, recognized by Abs that bind to epitopes not exposed in the context of the functional trimer; (ii) the neutralizing face, which binds most known NAbs; and (iii) the silent face which is composed of variable determinants, is heavily glycosylated and thus is poorly immunogenic [141–143]. Concerning influenza virus, N-glycosylation of the hemagglutinin (HA) and neuraminidase surface proteins is of major importance in biosynthesis, stability, virus release, receptor binding, infectivity and neurovirulence [139, 144]. The number of N-glycosylation sites in HA, the major target of influenza virus NAbs, reportedly ranges from 5 to 11 [139]. Glycans located in the stalk region of the HA contribute to the folding, stability, trimerization and transport of HA molecules to the cell surface and thus are highly conserved. In addition, it is well established that glycans located at HA globular head can modulate the immune recognition of influenza viruses by masking or modifying antigenic sites.

Intriguingly, although HCV, HIV and the influenza virus share the common feature of shielding neutralizing epitopes with glycans, some differences related to the evolution of the glycan shield have been detected [140]. Indeed, variation in the number and location of glycosylation sites in the globular head of influenza HA could arise by antigenic drift, in order to prevent recognition of epitopes targeted by NAbs elicited by previously circulating strains. For instance, it has been observed that the number of sequons (*i.e.*, potential glycosylation sites) in HA in the pandemic H3N2 virus increased from 6 to 10 between 1968 and 2000, which makes it more refractory to Abs recognition [145]. For HIV, modification of the glycan shield under immune pressure in each infected patient seems to prompt an immune escape mechanism that allows the virus to persist despite the presence of an evolving Ab repertoire [143]. Apparently, there is immune-mediated selection pressure to both change the position of individual glycans and conserve gp120 overall glycan shield, since mutations that both destroy and create N-glycosylation sites are selected. Two kinds of sequons have been evidenced in HIV: (i) fixed sites (embedded in readily aligned positions); and (ii) shifting sites that shift in relative position and regional density due to point mutations, insertions or deletions [140]. Of course, this evolution is driven by the need to protect the adjacent, neutralizing epitopes. However, it may also be a mechanism to continually destroy non-self patterns formed by multiple glycans, which would be interpreted as a danger signal by the innate and adaptive immune systems as illustrated by the existence of the monoclonal NAb 2G12, which recognizes a cluster of glycans on gp120 silent face. Although HCV is an extraordinarily variable virus, N-glycosylation sites in HCV E1E2 proteins are far less variable than in HIV gp120 and influenza HA. Indeed, 13 of the 17 potential sequons are found in almost 100 % of E1E2 sequences [129]. Furthermore, only two shifting sites have been observed in E2, E2N5 and E2N9 [140], which are located in HVR2 and the intergenotypic variable region (IgVR), respectively (Figure 1) [146,147]. Hence, in contrast to HIV and influenza, the HCV glycan shield does not appear to be evolving.

Taken as a whole, these data emphasize the importance of targeting carbohydrate antigens on the virus surface in vaccine and/or therapeutic strategies. It has long been thought that the glycosylation status of viral envelope proteins can explain the poor immune control by hosts in several infectious diseases. Many studies have suggested that understanding the role of glycosylation is critical for: (i) defining the virological properties and immunogenicity of viral envelope proteins; and (ii) designing carbohydrate mimetics that could be used as immunogens in vaccine development [148,149]. Understanding the three-dimensional organization of the sugars on viral envelope proteins could guide the selection of mutants with the fewest antigenic and functional alterations but with enhanced neutralization sensitivity. Furthermore, it is now well-established that carbohydrate binding agents can inhibit viral entry of numerous viruses including HCV by binding to envelope protein glycans and preventing interactions between viral envelope proteins and specific cell surface receptors [150–152]. Pradimicin A is of particular interest for the development of new therapeutic strategies, as it is a small, non-peptidic compound that is likely to be less immunogenic and more stable than the lectins [151,153]. These observations suggest that it will be possible to design small antiviral molecules that target viral envelope protein glycans. Resistance to this type of drug is likely to develop and will probably result in mutations at some glycosylation sites and thus come at a replicative cost to the virus, as observed for HIV [154–156]. It has been argued that this strategy could be very efficient, since these compounds could not only directly inhibit viruses, but also induce partial loss of the glycan shield and make the virus more vulnerable to attack by the immune system [157,158].

## Figures and Tables

**Table 1.** The main anti-HCV monoclonal NAbs with available mapping information.

**Monoclonal NAb**	**Specificity**	Epitope *	Residues Involved in Epitope Formation *	**References**
**Anti-E1**				
IGH505	Cross-reactive	313–326	316, 320, 323	[50,51]
IGH526	Cross-reactive	313–326	316, 319, 320, 323, 324	[50,51]
H-111	Cross-reactive	192–202	195, 196, 198, 199, 201	[52,53]

**Anti-E2**				
3C7	H strain	HVR1–396–407		[54]
9/27	H strain	HVR1–396–407 (partially conformation-dependent)		[19,55]
AP213	Gla strain	HVR1–396–407 (partially conformation-dependent)	400, 403, 404	[56]
J6.36	J6 strain	HVR1 (partially conformation-dependent)	403, 406	[57]
3/11	Cross-reactive	412–423 (partially conformation-dependent)	415, 420, 421	[19,55,58,59]
AP33	Cross-reactive	412–423 (partially conformation-dependent)	413, 415, 418, 420	[58,59]
HCV1	Cross-reactive	412–423	413, 420	[60]
H77.39	Cross-reactive	Located between 384–520	415, 417	[57]
1:7	Cross-reactive	Conformational	523, 529, 530, 535	[23]
A8	Cross-reactive	Conformational	523, 529, 530, 535	[23]
CBH-2	Cross-reactive	Conformational	425, 426, 431, 523, 529, 530, 535	[61]
CBH-5	Cross-reactive	Conformational	523, 525, 530, 535	[62]
CBH-7	Cross-reactive	Conformational	549	[62]
HC-1	Cross-reactive	Conformational	529, 530, 535,	[61]
HC-12	Cross-reactive	Conformational	530, 535	[61]
AR3A	Cross-reactive	Conformational	424, 523, 525, 530, 535, 540	[63]
AR3B	Cross-reactive	Conformational	424, 530, 535	[63]
AR3C	Cross-reactive	Conformational	424, 525, 530, 535, 540	[63]
AR3D	Cross-reactive	Conformational	424, 530	[63]
H35	Poorly cross-reactive	Conformational	523, 527, 529, 530, 535, 550	[12,48]
H48	Poorly cross-reactive	Conformational	523, 529, 530, 535, 550	[12,48]

* The numbers correspond to the positions in the polyprotein of the reference strain H (GenBank accession no. AF009606).

**Table 2.** Summary of the properties of glycosylation mutants.

**Virus**	**HCVcc Infectivity^a^**	**HCVpp Infectivity^a,b^**	**Core Release^c^**	**Sensitivity to Neutralization^d^**
Wild-type	+++	+++	++	+
Mutant				
E1N1	+/−	++	−	ND (+)
E1N2	++	+	+	ND (+)
E1N3	+++	++	++	ND (+)
E1N4	++	+	+/−	ND (+)
E2N1	+++	++	++	++
E2N2	++	− (−)	++	++^e^
E2N3	+	+++	+	ND (+)
E2N4	++	− (−)	+	++^e^
E2N5	++	++	++	+
E2N6	+++	++	++	++
E2N7	+/−	+++ (+)	+	ND (−)
E2N8	−	−	+/−	ND
E2N9	+++	+++	++	+
E2N10	−	−	−	ND
E2N11	+	+	+/−	++

a Percentage of infectivity: +++, >90%; ++, between 30% and 90%; +, between 10% and 30%; +/−, between 2% and 10%; −, <2%.

b Infectivity of HCVpp of genotype 1a, as previously reported [130]. The values in brackets are the results obtained for genotype 2a HCVpp.

c Percentage of core release: ++, >75%; +, between 30% and 75%; +/−, between 12% and 30%; −, <12%.

d Sensitivity to antibody neutralization: +, similar to wild-type; ++, more than a 5-fold increase in sensitivity to neutralization with most antibodies tested; − decrease in sensitivity to neutralization. The values in brackets were obtained for genotype 1a HCVpp only [129]. ND, not determined.

e Results obtained with the HCVcc system only.
